# Complex Pattern of Platelet Activation/Reactivity After SARS-CoV-2 Infection

**DOI:** 10.3390/ijms26010049

**Published:** 2024-12-24

**Authors:** Boguslawa Luzak, Jacek Golanski, Marcin Rozalski

**Affiliations:** Department of Hemostasis and Hemostatic Disorders, Medical University of Lodz, Mazowiecka 6/8, 92-215 Lodz, Poland; jacek.golanski@umed.lodz.pl (J.G.); marcin.rozalski@umed.lodz.pl (M.R.)

**Keywords:** blood platelets, COVID-19, platelet reactivity, SARS-CoV-2 spike protein, thrombotic complications, anti-SARS-CoV-2 antibodies

## Abstract

COVID-19 and post-COVID (long COVID) are associated with thromboembolic complications; however, it is still not clear whether platelets play a leading role in this phenomenon. The platelet hyperreactivity could result from the direct interaction between platelets and viral elements or the response to inflammatory and prothrombotic factors released from blood and vessel cells following infection. The existing literature does not provide clear-cut answers, as the results determining platelet status vary according to methodology. Elevated levels of soluble markers of platelet activation (P selectin, PF4), increased platelet aggregates, and platelet-derived microparticles suggest the activation of platelets circulating in the bloodstream of COVID-19 patients. Similarly, platelets isolated from COVID-19 patients demonstrate increased reactivity in response to collagen, thrombin, and ADP. By contrast, an analysis of whole blood from COVID-19 patients indicates the reduced activation of the fibrinogen receptor. Similarly, some in vitro studies report potential targets for SARS-CoV-2 in platelets, whereas others do not indicate any direct effect of the virus on platelets. The aim of this work is to review and evaluate the reliability of the methodology for testing platelet function after contact with SARS-CoV-2. Despite the diversity of methods yielding varying results and the influence of plasma components or blood cells, it can be concluded that platelets play an important role in the development of thrombotic complications after exposure to SARS-CoV-2.

## 1. Introduction

During COVID-19, endothelial cells or blood cells (platelets, leukocytes, and red blood cells) might interact with the SARS-CoV-2 virus or any of its components, leading to thromboembolic consequences. Months after SARS-CoV-2 infection, the dysregulation of platelet function and thrombotic problems have also been linked to post-COVID (long COVID) syndrome. The phenomenon of platelet hyperreactivity in COVID-19 patients, particularly those with a severe course of the disease, is well known, but it is impossible to determine with certainty whether it is caused by the prothrombotic and inflammatory factors released from both blood and vessel cells or by direct interaction between platelets and viral elements. A detailed analysis of the literature does not provide clear-cut answers, as the results determining platelet status vary according to methodology. Increased levels of platelet aggregates, platelet-derived microparticles, and soluble indicators of platelet activation (P selectin, PF4) suggest the activation of platelets circulating in the bloodstream of COVID-19 patients, while aggregation tests suggest increased platelet reactivity in response to agonists (collagen, thrombin, ADP). However, studies on whole blood from COVID-19 patients show that the fibrinogen receptor (GPIIbIIIa, α2bβ3) is less activated. In addition, lower GPIIbIIIa activation and platelet aggregation has been noted in whole blood in the presence of the SARS-CoV-2 spike protein and anti-SARS-CoV-2 neutralizing antibodies, which may suggest that the spike protein/anti-SARS-CoV-2 antibody complex has a part in this process [1].

A key role in the prothrombotic complications observed in COVID-19 patients was believed to be played by platelet hyperactivation, but recent publications suggest it may be more closely linked to SARS-CoV-2 infection [2]. Additionally, antiplatelet therapy, surprisingly, does not lead to clear clinical benefits in COVID-19 patients. Recently, COVID-19 has been suggested to represent *an endothelial disease* [3]. Endothelial disruption and a cytokine storm result in a prothrombic state, including the stimulation of platelets and other blood cells; in addition, it has been found that the SARS-CoV-2 virus or its proteins are internalized by platelets, suggesting the possibility of direct platelet–virus interaction.

This review provides a summary of recent findings concerning the monitoring of blood platelet function during COVID-19 infection and in vitro studies when SARS-CoV-2 or its components are present. One reason for the wide variation in findings among studies is that platelet activation and blood hypercoagulation may be caused by a wide range of factors and mechanisms rather than SARS-CoV-2 itself. Also, the literature uses a wide range of methods for testing the function of platelets affected by SARS-CoV-2, and some do not accurately reflect their physiological function.

## 2. Overview of Methods for Blood Platelet Activation/Reactivity Monitoring

The most recognized physiological role of blood platelets is their contribution to hemostasis, i.e., preventing blood loss after vascular injury; however, in pathological conditions, platelet activation leads to thrombosis in arterial or venous circulation and prevention in microcirculation. Following a stimulus (e.g., vascular injury, activated endothelium), platelets demonstrate adhesion and immobilization on collagen through vWF, aggregation, activation, and granule release; this is accompanied by enhanced platelet activation via thromboxane A2 initiated by COX-1 and ADP release, as well as the activation of fibrinogen receptor (αIIbβ3; GPIIbIIIa), prothrombinase complex formation, and thrombin generation. The consequence of the activation and release reaction is the appearance of markers such as P selectin (CD62P) and CD40L (also known as CD154) on the platelet surface. In addition, substances released from platelet granules serve as plasma-soluble markers of platelet activation. Among them, the most common are platelet factor 4 (PF4), also known as CXC chemokine ligand 4 (CXCL4), P selectin (sCD62), thromboxane B2, thromboglobulin, or thrombomodulin [4]. The exposure of these proteins on the platelet surface, including P selection and CD40L, promotes further interaction between platelets and other blood cells and the generation of aggregates. Blood platelets and megakaryocytes are also the primary source of extracellular vesicles (EVs) in the blood circulation; these can be considered as biologic markers of the state of platelet activation [4].

**Figure 1:**
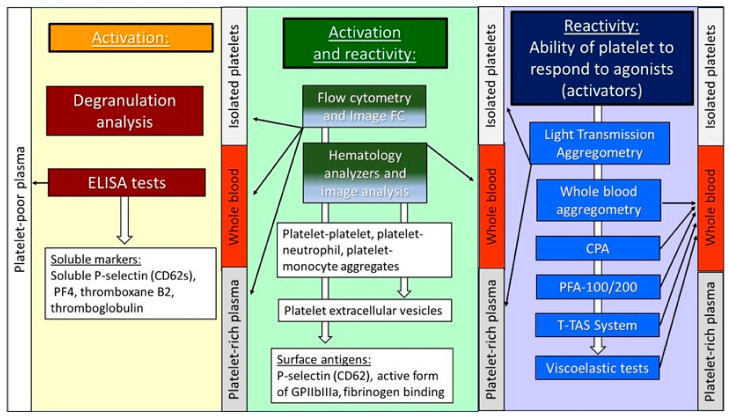
The most commonly used methods for the evaluation of human blood platelet function, involving activation and reactivity. The platelet function may be measured in whole blood, platelet-rich plasma, and isolated platelet suspension, as well as in platelet-poor plasma for the testing of soluble markers. ELISA—enzyme-linked immunosorbent assay; PF-4—platelet factor 4; PFA—platelet function analyzer; T-TAS—Total Thrombus formation Analysis System; cone and plate (let) analyzer.

The tests used to study platelet activation and reactivity are comprehensively reviewed by Larsen et al., who discuss also the methodology used to test platelet function in COVID-19 patients [5]. The platelet function tests are well established, and their methodological features and clinical applications are described by Paniccia et al. [6] and Michelson et al. [7]. While the terms *platelet activation* and *platelet reactivity* are used interchangeably in most publications, a clear distinction should be drawn between the two terms, because platelets activated in the vascular bed are generally less reactive [8]. The most commonly used methods for the evaluation of human blood platelet function are described below and shown in Figure 1.

### 2.1. Platelet Aggregometry

#### 2.1.1. Light Transmission Aggregometry (LTA)

Light transmission aggregometry is the most popular method for measuring aggregation and is considered the gold standard in testing platelet reactivity [9]. Briefly, platelet aggregation is assessed by measuring the change in light transmittance after adding agonists to platelet-rich plasma (PRP) at the recommended concentration. Before analysis, the maximum light transmittance of the sample should be determined (100%) in platelet-poor plasma (PPP). Since LTA is the most widely used method for testing platelet aggregation, several attempts have been made to standardize the methodology between laboratories [10]. Unfortunately, LTA analysis requires a relatively large volume of blood, especially if several agonists are used, such as collagen, adenosine diphosphate (ADP), arachidonic acid (AA), and ristocetin for testing platelets from one individual [10,11,12]. LTA may be also used to monitor isolated platelets in suspension [13].

Two promising modifications of classical LTA aggregation have been described, these being 96-well plate-based aggregometry [5,9,14] and analysis using coagulation machines (e.g., Sysmex) [15]. The OPTIMUL assay uses standard half-area 96-well microtiter plates with seven commonly used platelet agonists (AA, ADP, collagen, ristocetin, epinephrine, TRAP, and U46619); the method was found to be more sensitive to the thromboxane pathway, and its results seem to be promising for screening/diagnosis, especially considering the multiple advantages over traditional LTA platforms [16].

#### 2.1.2. Whole Blood Aggregometry (WBA)

Aggregation can also be measured by whole blood aggregometry, which is based on the change in electrical resistance or impedance between two electrodes. This obviates the need to prepare a platelet suspension and allows platelet function to be measured under more physiological conditions. While platelet aggregation is performed in anticoagulated whole blood, most commonly with a reduced calcium concentration, platelet function testing takes place in the presence of other blood cells (leucocytes, erythrocytes) and plasma components. Whole blood aggregometry can be used to monitor platelet adhesion and aggregation, but it is not widely used because it provides no information on shape change or on platelet aggregation or disaggregation kinetics; these parameters are essential for accurately assessing platelet function defects [13]. Furthermore, the method needs platelet counts in the normal ranges and cannot be used in thrombocytophenic patients [17]. The results of this method are also influenced by the leukocyte count [5,8,18].

### 2.2. Flow Cytometry

Flow cytometric (FC) analysis has been widely used in studies of platelet function including multiple clinical settings, such as the diagnosis of inherited platelet disorders and the measurement of circulating platelet activation and/or reactivity [19]. Among the available platelet function assays, flow cytometry remains the superior approach to measure platelet function in thrombocytopenia.

#### 2.2.1. Surface Markers

Flow cytometry allows for the determination of changes in the expression of receptors, including P selectin, fibrinogen receptor GPIIbIIIa, von Willebrand (vWF) factor receptor GPIb/IX/V, and collagen GPVI [19,20,21]. In FC, fluorescence is measured for a single platelet, and the result is expressed either as mean fluorescence intensity or as a percentage (i.e., fraction of antigen-positive platelets). The method enables the design of panels with several fluorophores, facilitating the simultaneous measurement of many platelet activation markers. Surface markers can be measured on resting platelets or after activation with various agonists [11]. The FC tests can be performed in a very small volume of blood, and the results are not sensitive to lower platelet counts. Additionally, research protocols can be designed individually, allowing for great freedom in both research and clinical applications. The main advantage of this method is that a number of standardization and research procedures are available in the literature [5]. The most popular surface markers of platelet activation/reactivity are P selectin (CD62), the active form of GPIIbIIIa (PAC-1 binding), fibrinogen, or vWF binding.

#### 2.2.2. Detection of Aggregates and Procoagulant Platelets

Standard flow cytometry allows for a detection of platelet–neutrophil, platelet–lymphocyte, and platelet–monocyte aggregates and procoagulant platelets [22]. The results are expressed as the percentage of monocyte–platelet aggregates/neutrophil–platelet aggregates [19].

Procoagulant platelets are a subpopulation of platelets that promote coagulation by providing the procoagulant surface, enabling the assembly of coagulation factors and thrombin generation. This group can be detected using a combination of P selectin and annexin V (phosphatidylserine marker) [23]. The flow cytometry assessment of platelets using the combination of GSAO [4-(N-(S-glutathionylacetyl) amino)phenylarsonous acid], a dithiol-reactive probe, and P selectin, is a novel and powerful technique for identifying and quantifying the procoagulant subpopulation of platelets that can support thrombin generation [24].

#### 2.2.3. Imaging Flow Cytometry

A very promising direction for the development of FC is imaging flow cytometry (IFC), an emerging technology that combines conventional FC with microscopy to enable the analysis of heterogeneous cell populations with high throughput and high spatial resolution by acquiring images of single cells or cell aggregates [25]. Another interesting direction of research is the digital analysis of cytometric or microscopic images, which allows for the number of platelet and platelet–leukocyte aggregates to be determined [26,27].

Modern hematology analyzers are not much different from flow cytometers, but their main advantage is the easy analysis of the occurrence of platelet and platelet–leukocyte aggregates [28]; this possibility is further developed by digital image analysis [29]. Imaging tools are designed to observe diverse molecular and morphological changes in cells and/or dynamic interactions within a network of living cells in vitro and in vivo [30].

#### 2.2.4. Platelet Extracellular Vesicles

Activated platelets release a heterogeneous population of extracellular vesicles (EVs), including microparticles, which are thought to mediate both inflammation and coagulation. Platelet-derived EVs are increased in a wide variety of inflammatory conditions, including autoimmune diseases, as well as cancer and viral infections [31]. Platelet activation is closely related to the occurrence of circulating procoagulant tissue factor (TF)-bringing extracellular vesicles [32]. Despite indications that circulating EVs may be promising biomarkers of variety of diseases, the procedure lacks standardized analytical methodology. The small size and heterogeneity of EVs make them undetectable with traditional methods, including conventional flow cytometers. The development of high-sensitivity flow cytometry (hsFCM) has enabled the detection of single EVs and their accurate sizing [31].

### 2.3. Platelet Adhesion and Blood Flow Methods

Currently, static methods are rarely used to assess platelet adhesion. The process of platelet adhesion to the exposed subendothelial matrix is a multistep one, influenced by the local shear rate of blood at venous flow. Platelets can interact with collagen (via GPVI and α2β1), fibronectin (via integrin αIIbβ3, αVβ3, and α5β1) and laminin (via α6β1) present in the extracellular matrix [33]. Platelet adhesion and thrombus formation can be monitored in microfluidic flow chambers on surfaces coated with adhesive proteins. These devices can characterize platelet function under flow with low blood volume requirements and controlled conditions. They can mimic the anatomy of healthy and stenotic blood vessels, recreate a range of physiological and pathological shear stress conditions, and be used to investigate platelet shape or platelet accumulation over different adhesive proteins [34].

### 2.4. ELISA Tests—Measurements of Release Reaction Markers in Plasma

Activated platelets release small biomolecules and more than 300 proteins known to regulate the hemostatic, inflammatory, and angiogenic responses of platelets, leukocytes, and vascular cells. Most of the platelet-released proteins are derived from granule cargos and proteolytically cleaved/shed membrane-bound proteins such as receptors and platelet-derived extracellular vesicles. The platelet-released proteins present in plasma and isolated platelets can be assessed qualitatively and quantitatively by advanced enzyme-linked immunosorbent assays (ELISAs) and mass spectrometry. The platelet α granule secretome covers the majority of released platelet proteins, which are synthesized in megakaryocytes or endocytosed from plasma. The α granules contain large adhesive proteins, such as vWF, thrombospondin-1 (TSP-1), vitronectin, and fibronectin, as well as various coagulation factors (factor V, VII, XI, XIII), mitogenic factors (platelet-derived growth factor (PDGF), vascular endothelial growth factor (VEGF), transforming growth factor β (TGF-β), protease inhibitors (protein C, PAI-1), tissue factor pathway inhibitor (TFPI), membrane proteins such as P selectin (CD62P) and CD40L, and various chemokines, including β-thromboglobulin (beta-tg) and PF4. Elevated levels of sP selectin, sCD40L, and soluble GPVI are mostly used as platelet activation markers. Activated platelets also release thromboxane A2 (TxA2), which is converted to TxB2 and measured in plasma as a marker of arachidonic acid metabolite [35]. Platelet granule secretion is often used as a first-line test for the assessment of platelet function because platelet reactivity may give normal results in patients [36].

### 2.5. Other Methods of Monitoring Platelet Responses to Stimuli or Combined Primary and Secondary Hemostasis

#### 2.5.1. Viscoelastic Tests

Viscoelastic tests include thromboelastography (TEG) and thromboelastometry (TEM); these are global coagulation testing methods that assess the physical properties of clot formation in a whole blood sample in vitro in real time [37]. Intensive work is underway to enrich classical methods with a version enabling the assessment of platelet function. Particularly interesting is the development of the TEG-PM (thromboelastography with platelet mapping) method [37].

#### 2.5.2. IMPACT: Cone and Plate (Let) Analyzer

Cone and Plate (Let) Analyzer acts by measuring platelet adhesion to a thrombogenic polystyrene surface under high shear stress. Whole blood is added to the surface and high shear stress is generated by a rotating cone. This stimulates platelets to adhere to the plasma proteins, particularly fibrinogen and vWF, on the stationary plate [5,7].

#### 2.5.3. Platelet Function Analyzer

The PFA-100 and PFA-200 (platelet function analyzer) flow analyzers are designed to assess the function of platelet-dependent primary hemostasis [38]. These analyzers, used to replicate natural conditions for testing platelet reactivity, originally used two types of measuring cassettes with a flow system simulating a damaged blood vessel; one version contains collagen and epinephrine, while the other includes collagen and ADP. Platelets in whole blood (collected with citrate) flowing through the capillary in the cassette are activated and cause the flow opening in the membrane to close, which is recorded by the analyzer as the occlusion time. In addition to platelet reactivity, the occlusion time is also influenced by hematocrit, platelet count, and von Willebrand factor concentration. The latest version of the analyzer is called INNOVANCE PFA-200 System and allows for the use of three types of measuring cassettes, namely Dade^®^ PFA Collagen/EPI Test Cartridge, Dade PFA Collagen/ADP Test Cartridge, and INNOVANCE^®^ PFA P2Y12. The system is constantly modified, and a new version has been introduced—PFA-200 [38].

#### 2.5.4. Total Thrombus Formation Analysis System

Many methods for testing platelet function based on microflow analysis have been described [39], but currently, the closest to laboratory diagnostics is the T-TAS System (Total Thrombus formation Analysis System). The system is somewhat similar to PFA, but the structure of the measuring cassettes is completely different. Whole blood collected for hirudin flows through the capillary system simulating blood vessels, which are coated with collagen (PL test) or collagen and tissue thromboplastin (AR test or HD test), respectively [40]. By using the T-TAS system, platelet thrombi are formed in primary or secondary conditions.

A detailed description of more rarely used methods not discussed in this work can be found on the UptoDate website (https://www.uptodate.com/contents/platelet-function-testing; accessed on 29 November 2024) or in other reviews [5,9,41].

## 3. Platelets in COVID-19

### 3.1. Platelet Count and Volume Change in COVID-19 Patients

In COVID-19 patients, platelet count and mean platelet volume (MPV) were extensively studied, and it was suggested that these parameters are important and predictive in COVID-19 [42,43,44]. In SARS-CoV-2 patients, the platelet count differs between mild and severe cases, but the mean platelet volume tends to be increased [45]. It has also been observed that the change in platelet count over the course of the disease would affect the prognosis of COVID-19. Platelet counts appear to be strong prognostic indicators of coagulation abnormalities in patients with severe COVID-19 [43,46]. A lower platelet count was found in half of patients infected with SARS-CoV-2 [43]. In individuals with mild symptoms, a significant drop in the number of platelets was usually a self-limiting condition, with platelet counts typically returning to normal within two weeks [43,46]. Mean platelet volume was significantly higher in COVID-19 patients compared to non-COVID-19 patients [47] and differs between mild and severe cases [44]. Wolny et al. showed that in severely ill patients suffering from COVID-19, the platelet count was lower and the immature platelet level was higher than in less severely ill patients [48]. Immature platelets are a fraction of large, young platelets; hence, it is obvious that increased values are observed in patients with severe COVID-19 [42,44]. Elevated MPV in the circulation may indicate a larger proportion of young platelets, which could result from a decrease in the number of platelets during the course of the disease [49]. In addition, platelet distribution width (PDW) significantly correlates with COVID-19 infection severity and mortality [50].

### 3.2. Platelet-Related Biomarkers

Although platelet parameters alone may not accurately reflect the severity of COVID-19, they can provide important information about disease severity when combined with leucocyte parameters and blood cell count ratios [46]. Various versions of parameters related to blood cells are used in the literature (Table 1).

The platelet-to-lymphocyte ratio (PLR) is easily obtained from complete blood count panels. Recently, it has been proposed as a better indicator of inflammation than white blood cell count alone. The PLR was suggested as a useful biomarker to predict the severity of COVID-19 in patients [51]. In a systematic review and meta-analysis, Simadibrata et al. report that a higher level of PLR on admission in COVID-19 patients is associated with increased morbidity and mortality [52]. In addition, the systemic inflammatory index (SII) and systemic inflammatory response index (SIRI) have been described as being of prognostic importance in COVID-19 patients [53]; indeed, both the SII and SIRI were found to predict disease severity and mortality in patients with COVID-19, with high SII and SIRI levels indicative of a poor prognosis. The authors suggest that the SII and SIRI are easily accessible and inexpensive indices in the emergency department and can be used as auxiliary tests for prognosis prediction [53].

### 3.3. Determination of Platelet Activation/Reactivity Status in SARS-CoV-2 Patients

The results from studies on platelet reactivity in COVID-19 vary significantly, depending on patient characteristics, disease status, control group characteristics, and experimental protocols. Table 2 summarizes the representative studies on platelet activation/reactivity in COVID-19 patients.

Several studies show an elevated blood platelet activation and the alteration of platelet count in COVID-19 patients [54,55,56,57]. Platelets from subjects with severe forms of SARS-CoV-2 infection possess an increased surface exposure of CD62P [56] and activated GPIIb/IIIa complex [57]. Also, a greater release of thromboxane A2 [58] and elevated platelet–leucocyte aggregate formation was observed [56]. Manne et al. report that platelets from COVID-19 patients had increased P selectin expression both at baseline and upon activation with TRAP or 2MeSADP [58]. They also found that circulating platelet–neutrophil, platelet–monocyte, and platelet–T-cell aggregates were significantly elevated in COVID-19 patients compared with healthy donors. Furthermore, significantly higher platelet aggregation was noted in COVID-19 patients in response to low-dose agonists (2MeSADP, thrombin, and collagen), and platelets showed excessive spread on both fibrinogen and collagen [58]. Zaid et al. demonstrated that platelets were hyperreactive in non-severe and severe COVID-19 patients, with aggregation occurring at suboptimal thrombin concentrations. Furthermore, the platelets adhered more efficiently onto collagen-coated surfaces under flow conditions [26].

Canzano et al. suggested that the cytokine storm present in COVID-19 patients induces massive cell activation with the production of tissue factors, mainly by platelets, granulocytes, and EVs, in addition to altering the balance of endothelial function. They also reported that COVID-19 plasma, added to the blood of healthy subjects, induces platelet activation similar to that observed in vivo in COVID-19 patients [59]. Also, Zlamal et al. reported that IgG antibodies from patients with severe COVID-19 are able to stimulate FcƴRIIA, leading to the induction of procoagulant platelets with increased potential for thrombus formation [60]. Pelzl et al. found that incubation of healthy platelets with sera or IgG from COVID-19 patients increased the generation of procoagulant platelets and that this is mediated by IgG antibodies through the PI3K/AKT signaling pathway in an FcγRIIA- dependent manner [61]. Puhm et al. reported that platelets are not activated by SARS-CoV-2 nor purified spike proteins but rather by TF derived from extracellular vesicles released from monocytes. Similarly, it appears more likely that during COVID infection, platelet hyperactivation is caused by inflammatory stress associated with low concentrations of SARS-CoV-2 in the circulation via direct virus–platelet interaction [62].

Although hypercoagulability is one of the main characteristics of COVID-19, several papers report reduced platelet reactivity in SARS-CoV-2 patients [58,63,64]. Ruperto et al. found lower platelet aggregation in platelet-rich plasma in response to both ADP and collagen in COVID-19 patients than in healthy volunteers; however, no significant differences in platelet aggregation were observed in response to arachidonic acid [63]. Manne et al. found that in whole blood from COVID-19 patients, platelet activation by 2MeSADP, TRAP, and collagen-related peptides (CRPs) resulted in decreased PAC-1 binding, irrespective of disease status, compared to healthy donors. The authors suggest that the decreased PAC-1 binding was not due to changes in αIIb expression, since the latter did not differ significantly between healthy donors and all COVID-19 patients [58]. Heinz et al. found that TRAP or arachidonic acid did not influence platelet aggregability in COVID-19 patients compared to healthy subjects, whereas it was reduced by ADP treatment; however, they concluded that their results were limited by the small group size [65].

These examples of lower platelet reactivity may be explained by the fact that platelets could be extensively activated in vivo during COVID-19; this may result in refractoriness to new agonists added during ex vivo platelet function tests, a phenomenon called exhausted platelets [64]. Another hypothesis is that platelet reactivity may be modulated by serum/plasma components from COVID-19 patients and by IgG anti-SARS-CoV-2 antibodies [59,60,66]. Althaus et al. report that sera from COVID-19 patients induced a significant increase in apoptosis markers (mitochondrial inner transmembrane potential, cytosolic Ca^2+^, and PS externalization) compared with healthy volunteers. Interestingly, immunoglobulin G fractions from COVID-19 patients induced an Fcƴ receptor IIA–dependent platelet apoptosis [67]. A recent proteomic analysis of platelets indicated that agonist–induced phosphatidylserine exposure and integrin αIIbβ3 activation were impaired in COVID-19 patients, and that COVID-19 led to maximal levels of P selectin–dependent platelet–neutrophil aggregates [68]. Bakowski et al. reported the reduced, below the reference range for all tested agonists, whole blood platelet aggregation in acute COVID-19 patients (Table 2). In addition, on day 1, median aggregation was significantly lower in non-survivors compared to survivors. On days 3 and 5, platelet response to agonists remained below normal, with significantly lower values in non-survivors compared to survivors. Simultaneously, this observation was consistent with the results of the platelet counts in blood. On day 1, all patients had platelet counts within the reference range. In survivors, the minimum and maximum platelet counts were 169.0–553.0, 132.0–406.0, and 106.0–302.0 × 10^9^/L, and in non-survivors, the counts were 137.0–418.0, 105.0–344.0, and 102.0–314.0 × 10^9^/L on days 1, 3, and 5, respectively [69]. Wolny et al. have also shown impaired platelet function in COVID-19 patients treated with intubation and extracorporeal membrane oxygenation (ECMO) [48].

An interesting comprehensive platelet function study was reported by Weiss et al. [70]. They compared platelet function assessed by flow cytometry in 37 COVID-19 patients (46 with sepsis, 28 without) with 35 healthy control participants and found that patients with sepsis showed markedly reduced α granule release upon stimulation compared to COVID-19 patients. In contrast, GPIIb/IIIa activation and δ granule secretion were profoundly deficient in critically ill patients with both COVID-19 and sepsis. In a microfluidic flow chamber model, thrombus formation was found to be severely impaired only in sepsis patients, while COVID-19 patients showed numerous and overall stable thrombi. These data strongly imply the presence of a SARS-CoV-2-specific dysfunctional platelet phenotype with blunted GPIIb/IIIa activation, which is uncoupled from functional α granule release in COVID-19 patients differing from bacterial sepsis [70].

**Table 2:** Analysis of platelet activation/reactivity in COVID-19 patients—examples from the literature.

Study Groups	Material/Method	Agonist to Platelet Activation	Results in COVID-19 Cases	Ref.
Non-ICU (n = 24) and ICU (n = 17) COVID patients vs. healthy controls (n = 17)	whole blood/flow cytometry	no agonist	increased P selectin expression (*p* < 0.05)	Manne et al. [58]
	PPP/ELISA	no agonist	increased level of P selectin (*p* < 0.05) and PDGF (*p* < 0.05)	
	whole blood/flow cytometry	2MeSADP (1 ng/mL), TRAP (2.5 µM)	elevated platelet–neutrophil (*p* < 0.001), platelet–monocyte (*p* < 0.001), and platelet–T-cell aggregates (*p* < 0.05); decreased PAC-1 binding (*p* < 0.05)	
	washed platelets/LTA aggregation	2MeSADP (5 nM or 50 nM), thrombin (0.05 U/mL or 0.5 U/mL), or collagen (2 µg/mL or 10 µg/mL)	increased aggregation induced by 2MeSADP (5 nM, *p* < 0.05 50 nM, *p* < 0.001), thrombin (*p* < 0.001), collagen (*p* < 0.05)	
	washed platelets/adhesion	collagen, fibrinogen	greater adhesion and spreading for collagen (*p* < 0.05) and fibrinogen (0.001)	
COVID patients (n = 18) vs. healthy controls (n = 9)	washed platelets/LTA aggregation	thrombin (0.05 U)	increased aggregation (*p* < 0.0001)	Zaid et al. [26]
	washed platelets/adhesion	collagen	greater adhesion (*p* < 0.001)	
Mild and moderate (n = 184) or severe COVID-19 cases (n = 57) vs. healthy controls (n = 166)	washed platelets/flow cytometry	thrombin (0.025 U/mL), collagen (0.6 µg/mL)	increased platelet integrin αIIbβ3 activation (PAC-1 binding) and P selectin (CD62P) expression (*p* < 0.01 for both markers)	Zhang et al. [71]
Acute COVID-19 patients (n = 11) vs. healthy controls (n = 11)	washed platelets/flow cytometry	no agonist	similar levels of annexin V-positive procoagulant platelets (NS)	Denorme et al. [72]
	washed platelets/flow cytometry	thrombin (1.0 U/mL) and convulxin (250 ng/mL) ionophore A23187 (50 µmol/L)	reduced level of annexin V-positive platelets (*p* < 0.0001); similar levels of PS exposure (annexin V binding) (NS)	
COVID patients (n = 60) vs. healthy controls (n = 60)	whole blood/aggregation (Multiplate^®^)	AA (0.5 mM; ASPI test), TRAP (32 µM), ADP (6.4 µM)	reduced aggregation for AA and TRAP (*p* < 0.01 for both agonists), without effect for ADP (NS)	Bertolin et al. [64]
COVID patients (n = 27) vs. healthy controls (n = 12)	whole blood/aggregation (Multiplate^®^)	AA (0.5 mM; ASPI test), TRAP (32 µM), ADP (6.4 µ)	lower results for mean AUC for ADP (*p* = 0.043; however, insignificant after age stratification—NS), no effect for AA or thrombin (NS)	Heinz et al. [65]
CAD-COVID patients (n = 55) vs. healthy controls (n = 39) or vs. CAD patients (n = 28)	whole blood/flow cytometry	no agonist	enhanced fraction of P selectin-positive platelets (*p* < 0.05) and fraction of platelet–leucocyte aggregates (*p* < 0.001)	Langnau et al. [56]
Moderate and severe COVID-19 cases (n = 46) vs. healthy controls (n = 29)	washed platelets/flow cytometry	no agonist	enhanced P selectin- or CD63-positive platelet fraction (*p* < 0.00001 for both markers); higher level of CD41/TF-bearing EVs (*p* < 0.00001)	Martins-Goccalves et al. [73]
	washed platelets/flow cytometry	thrombin (0.05 U/mL; 0.5 U/mL)	enhanced P selectin- (*p* < 0.00002) or CD63-positive platelet fraction (*p* < 0.00001)	
	washed platelets/ELISA	no agonist, 30 min incubation at 37 °C	enhanced level of PDGF (*p* < 0.002), RANTES (*p* < 0.00001), sCD62P (*p* < 0.00001)	
	PPP/ELISA	no agonist	enhanced level of sCD62P, PDGF, RANTES, PF4, TxB2 (*p* < 0.00001 for all the markers but TxB2 *p* < 0.006)	
	washed platelets/adhesion	fibrinogen	increased spread to fibrinogen-coated surface (without analysis of statistical significance)	
COVID (n = 37) vs. sepsis (n = 46) vs. infection patients (n = 28) vs. control (n = 35)	whole blood/flow cytometry	no agonist	no significant differences in PAC-1 binding (NS), higher *p* selectin exposure in COVID patients (*p* < 0.0001 vs. controls)	Weiss, et al. [70]
	whole blood/flow cytometry	ADP (5 μM); TRAP (5 μM)	GPIIb/IIIa activation (PAC-1 binding) markedly downregulated and CD62P exposure decreased in COVID-19 platelets vs. controls (*p* < 0.0001 for both markers)	
	recalcified whole blood/thrombus formation under venous shear (200 s^−1^) recalcified whole blood/thrombus formation under arterial shear (1000 s^−1^)	collagen, collagen, and TF collagen, collagen, and TF	platelet aggregation, thrombus volume, morphology, and contraction score were comparable between groups for collagen + TF (NS) and markedly reduced in COVID-19 vs. control for collagen (*p* < 0.0001) stable thrombus formation in COVID-19 patients, thrombi increased in number but smaller in individual size vs. control (*p* < 0.05); without external TF, no differences in platelet aggregation and thrombus volume in COVID-19 patients vs. control (NS)	
	whole blood/flow cytometric	no agonist, TRAP (5 μM)	markedly impaired dense granule release (reduced mepacrine uptake) vs. control (*p* < 0.0001)	
ICU COVID-19 patients (n = 13) vs. day controls	PRP/LTA aggregation	ADP (4 µmol/L), collagen (2 µg/mL), epinephrin (8 µmol/L), or ristocetin (1.2 mg/mL)	impaired agregability for ADP in 72%, collagen in 92%, ephinephrin in 55%, ristocetin in 17% of all cases	Kalbhen et al. [74]
	PRP/flow cytometry	thrombin (0, 0.05, 0.1, 0.2, 0.5, and 1.0 U/mL) with 1.25 mM GPRP; ristocetin (0.5–1 mg/mL); ADP (0.25, 0.75, 2.0 µM)	decreased CD62 and CD63 expression (*p* < 0.001 for both markers), impaired vWF binding in 12 of 13 COVID-19 patients, unaffected fibrinogen binding (NS)	
Severe (n =27) and moderate (n =9) COVID-19 patients vs. healthy controls (n = 15)	whole blood/image-based flow cytometry	no agonist	significantly higher platelet aggregate formation in both moderate (*p* = 0.0046) and severe (*p* < 0.0001) COVID-19 patients vs. control; more platelet aggregates in fatal COVID-19 cases compared to survivors (*p* < 0.0285)	Klenk et al. [27]
Acute COVID-19 patients treated with ECMO: survivors (n = 13) and non-survivors (n = 15)	whole blood/aggregation (Multiplate^®^)	AA (0.5 mM), TRAP (32 µM), ADP (6.4 µM), ristocetin (0.77 mg/mL)	platelet aggregation in most patients below the reference range for all tested agonists; significantly lower aggregation in non-survivors compared to survivors (*p* < 0.02 for AA; *p* < 0.05 for TRAP; *p* < 0.03 for ADP; *p* < 0.03 for ristocetin)	Bakowski et al. [69]
Mild, moderate/severe COVID-19 patients (n = 63) vs. healthy controls (n = 67)	whole blood/automatic hematology analysis (CELL-DYN Sapphire Hematology System)	no agonist	high level of platelet clumps with maximum at 10th day and persisted for 40 days; unchanged platelet count on first day (NS), rising on 5th day, 10th, and 40th (*p* < 0.01 for all days) compared to the reference group; elevated MPVs on first day (*p* < 0.05), gradually declining to reference level by 40th day	Nara et al. [28]

PDGF—platelet-derived growth factor; TRAP (thrombin receptor-activating peptide); CAD—coronary artery disease; AA—arachidonic acid; AUC—area under curve; ICU—intensive care unit; PPP—platelet-poor plasma; PRP—platelet-rich plasma; TF—tissue factor; GPRP—Gly-Pro-Arg-Pro; ECMO—*extracorporeal membrane oxygenation*.

### 3.4. Monitoring of vWF Binding to Platelets

A particularly interesting issue regarding platelet function in COVID-19 patients concerns the role of vWF. COVID-19 patients demonstrate significantly elevated levels of antigen and vWF activity, as well as ADAMTS-13 activity [75,76,77]. In addition, autopsies of COVID-19 patients who died of acute respiratory distress syndrome revealed numerous intrapulmonary arteriole thrombi, including fibrin, CD61-positive platelets, and megakaryocytes, with positive immunostaining of vWF [78]. In addition, mictrothrombi rich in platelets and vWF were more common in COVID-19 patients [79]. The relationship between platelets and vWF is very important in primary hemostasis. In platelets, vWF may not only bind to GPIbα (CD42b), a part of the GPIb-IX-V complex, but also to GPIIbIIIa, the receptor for fibrinogen, thus resulting in platelet adhesion, aggregation, and clot formation [75]. Ruberto et al. [63] reported markedly increased levels of vWF antigen and the vWF active form binding to platelets (vWF:RCo) in COVID-19 patients. These results were associated with higher ristocetin-induced agglutination rates, suggesting an increased capability of vWF to bind to platelets [63]. In the case of severe COVID-19, platelets were seen to bind more easily to plasmatic vWF, but no correlation was found between ristocetin sensitivity and vWF concentration in patient blood [80]. The opposite was observed by Kalbhen et al., who noted an impaired binding of vWF to platelets from COVID-19 patients after incubation with ristocetin; they also noted substantially higher vWF antigen concentration (vWF:Ag) than the normal range and elevated vWF collagen binding capacity (vWF:CB) [74].

The potential mechanism of the increased platelet–vWF interactions observed in COVID-19 patients or in vitro is difficult to explain. It is believed that the SARS-CoV-2 spike protein may interact with GPIbα and facilitate vWF-GPIbα binding. Li et al. report that SARS-CoV-2 can activate platelets directly and identified GPIbα as its binding receptor, although the binding affinity was only moderate [81]. They also indicated that RBD (spike receptor-binding domain) inhibited ristocetin-induced recombinant vWF binding on isolated platelets, which may suggest the competitive antagonism of GPIbα by the spike protein. However, Luzak et al. report that AK2 antibodies, which block vWF binding to GPIbα, reduced the plasmatic vWF binding to platelets in ristocetin-treated blood, irrespective of the presence of spike protein. These results indicate that spike protein interacts with platelets at a different site to the vWF binding site in platelet membrane GPIbα [1].

## 4. Effects of SARS-CoV-2 on Platelet Reactivity In Vitro

### 4.1. Structure and Active Domains in SARS-CoV-2

SARS-CoV-2 consists of a single strand of positive RNA that codes for four major structural proteins, namely the spike (S), membrane (M), envelope (E), and the nucleocapsid (N) protein. The virus infects host cells by engaging with its receptor, angiotensin-converting enzyme 2 (ACE2). The spike protein is the key factor for virus attachment to target cells [82]. This main SARS-CoV-2 membrane glycoprotein forms homotrimers that protrude from the viral surface; these two functional subunits facilitate viral attachment to the surface of the host cell (S1 subunit) and permit fusion of the viral and cellular membranes (S2 subunit). The distal part of the S1 subunit includes the receptor-binding domain (RBD) [83]. The spike protein contains an RGD motif (arginine–glycine–aspartate) near the distal tip of its receptor-binding domain, with structural features reminiscent of known integrin-binding proteins [84]. Since the spike protein of SARS-CoV-2 is involved in receptor recognition, as well as virus attachment and entry, it represents one of the most important targets for the development of SARS vaccines and therapeutics [85].

### 4.2. Presence of SARS-CoV-2 and Its Proteins in Blood

Although SARS-CoV-2 is a respiratory virus, multiple clinical manifestations suggest that this virus is present in tissues and body fluids [86,87] and can migrate from the lungs into the bloodstream. SARS-CoV-2 is more abundant in the circulation than previously thought, and it has been found that plasmatic viremia correlates with disease severity and mortality [88]. Varga et al. described the presence of SARS-CoV-2 elements within endothelial cells, with evidence of endothelial and inflammatory cell death during a post-mortem histological examination of affected tissues (kidney, lung, heart, liver) [89]. In addition to SARS-CoV-2 viral RNA, several studies show the presence of nucleocapsid antigen [90,91,92,93] or spike protein, either as a whole protein molecule or its S1 subunit in blood [88,94]. The presence of circulating spike protein in blood from the post-acute sequelae of COVID-19 patients (PACS) has been was reported for up to 12 months post-diagnosis, which may suggest that SARS-CoV-2 viral reservoirs may persist in the body [95]. Craddock et al. also found that 30% of PACS were positive for both spike and viral RNA, and a part of the circulating spike protein was linked to extracellular vesicles [96].

Additionally, Ogata et al. provided evidence that circulating SARS-CoV-2 proteins are present in the plasma of participants vaccinated with the mRNA-1273 vaccine from Moderna. The S1 antigen was detected as early as day 1 post-vaccination, and peak levels were detected on average five days after the first injection, with the mean S1 peak level being 68 pg/mL ± 21 pg/mL [97]. In another study, it was found that plasma from healthy donors contains circulating exosomes expressing spike protein on day 14 after vaccination with the mRNA-based SARS-CoV-2 vaccine (Pfizer-BioNTech); this was followed by a significant increase in spike protein level at day 14 of dose 2. The SARS-CoV-2 spike protein was also detected in exosomes after four months but at lower amounts [98]. Full-length SARS-CoV-2 spike mRNA vaccine sequences or their traces were found in blood up to 28 days after COVID-19 vaccination with Pfizer-BioNTech (BTN162b2) or the Moderna (mRNA-1273) mRNA vaccines [99]. Free spike protein antigen was detected in the blood of adolescents and young adults who developed post-mRNA vaccine myocarditis; also, interestingly, markedly elevated levels of full-length spike protein (33.9 ± 22.4 pg/mL), unbound by antibodies, were detected in the plasma of individuals with post-vaccine myocarditis, whereas no free spike protein was detected in asymptomatic vaccinated control subjects [100].

Various laboratories have detected SARS-CoV-2 RNA in isolated platelets in COVID-19 patients by RT-qPCR and virions in platelet sections by electron microscopy [26,58,71]. Zaid et al. reported the detection of SARS-CoV-2 RNA in 24% of patients with non-severe and 18% of patients with severe COVID-19 [26]. Additionally, Manne et al. observed mRNA from the SARS-CoV-2 N1 gene in platelets from 2 of 25 COVID-19 patients; the analysis revealed that these platelets lacked ACE2 receptor mRNA or protein, suggesting that they may have taken up SARS-CoV-2 mRNA independent of ACE2 [58]. Elsewhere, Koupenova et al. demonstrated the presence of a wide range of fragmented SARS-CoV-2 RNA in platelets from patients with COVID-19, direct platelet uptake of the virus, and the digestion of SARS-CoV-2 in platelets, making it non-infectious [101]. The internalization of the virus hence does not require ACE2, and its entry into platelets appears to occur by viral attachment to microparticles, some of which may be of platelet origin. Additionally, SARS-CoV-2 internalization triggers platelet death programs that cause platelet content to leak and subsequently reduce their functionality [101]. Bury et al. did not detect viral RNA in platelets from 24 COVID-19 patients [102].

### 4.3. Analysis of Blood Platelet Reactivity in the Presence of SARS-CoV-2 or Virus Componets

Direct virus–platelet interaction can occur in conditions in which circulating platelets are directly exposed to SARS-CoV-2, such as in severe cases of disease associated with viremia. Spike protein–platelet interaction is also possible shortly after anti-SARS-CoV-2 vaccination. The interactions between SARS-CoV-2 and blood platelets comprise the entry of the virus into platelets (virus internalization) or modulation of platelet function (platelet response to virus). Several model in vitro studies have attempted to elucidate such interactions. Most of these have been performed in washed (purified) human platelets incubated with spike protein or with SARS-CoV-2, such as S-pseudovirus expressing spike protein and cells such as Vero E6 or A549-hACE2, infected with SARS-CoV-2. The results of studies using whole blood or platelet-rich plasma underline the importance of the presence of anti-SARS-CoV-2 antibodies or viral proteins that could interfere with platelet stimulation assays [1,103].

The results from in vitro studies using whole blood, PRP, or washed platelets often indicate that the SARS-CoV-2 spike protein has no effect on platelets, not even a stimulatory effect (Table 3). A few reports describe the direct modulation of platelet function by SARS-CoV-2 or spike protein [62,71,81,104]. The spike protein significantly increases platelet activation/reactivity in an ACE2 receptor-dependent manner, reflected in PAC-1 binding, CD62P expression, α granule secretion, dense granule release, aggregation, platelet spreading, and clot retraction in vitro [71]. Li et al. reported that collagen-treated PRP samples incubated with spike protein demonstrated lower luminescence derived from ATP/ADP release compared to those treated with collagen alone [81].

Sevilya et al. compared the effect of several SARS-CoV-2 spike variants on platelet activation and found the platelet function to be dependent on the protein spike variant. In the study, engineered lentiviral particles were pseudotyped with spike SARS-CoV-2 variants and incubated with PRP obtained from healthy individuals. The pseudotyped SARS-CoV-2 exhibiting the wild-type Wuhan-Hu spike protein stimulated platelets to increase expression of the surface CD62P and activated αIIbβ3. The Delta SARS-CoV-2 variant clearly induced the highest levels of platelet activation, followed by the Wuhan-Hu. The Omicron BA.1 and Alpha variants induced the lowest levels of platelet activation. These results correlate with the clinical severity and mortality reported for these SARS-CoV-2 variants and may contribute to a detailed understanding of the molecular interactions involved in platelet activation in the COVID-19 disease [105].

**Table 3:** Studies of platelet function under in vitro conditions.

Protocol	Agonist/Method to Monitor Platelet Activation/Reactivity	Results from In Vitro Study	Ref.
incubation of washed platelets with SARS-CoV-2 (0.1 to 1 × 10^5^ PFU) for 30 min	no agonist/LTA aggregation	no change in platelet aggregation (NS)	Zhang et al. [71]
	collagen (0.6 μg/mL), thrombin (0.025 U/mL), ADP (5 μM)/LTA aggregation	increased platelet aggregation in SARS-CoV-2 dose-dependent manner (*p* < 0.01)	
	collagen (0.6 μg/mL), thrombin (0.025 U/mL)/LTA aggregation with luciferase	increased platelet dense granule secretion (ATP release) (*p* < 0.01)	
	no agonist or thrombin (0.025 U/mL)/flow cytometry	increased integrin αIIbβ3 activation (PAC-1 binding) and P selectin expression (*p* < 0.01)	
	fibrinogen/adhesion	enhanced spreading area after 40–60 min incubation (*p* < 0.01)	
	thrombin (1 U/mL)/clot retraction	enhanced clot retraction after 20–60 min (*p* < 0.05)	
incubation of washed platelets with spike protein (2 µg/mL, 5 min) or S1 subunit (2 µg/mL, 5 min)	no agonist/LTA aggregation	no change in platelet aggregation (NS)	
	collagen (0.6 μg/mL), thrombin (0.025 U/mL), ADP (5 μM)/LTA aggregation	elevated platelet aggregation (*p* < 0.05)	
	collagen (0.6 μg/mL), thrombin (0.025 U/mL)/LTA aggregation with luciferase	increased platelet dense granule secretion (ATP release) (*p* < 0.05)	
	no agonist or thrombin (0.025 U/mL)/flow cytometry	stimulation of platelets for PAC-1 binding and P selectin expression in the absence of agonist for spike protein (*p* < 0.01); increased PAC-1 binding and P selectin expression induced by thrombin for spike protein and S1 (*p* < 0.01)	
	fibrinogen/adhesion	enhanced spreading area after 40–60 min incubation (*p* < 0.01)	
	thrombin (1 U/mL)/clot retraction	enhanced clot retraction after 20–60 min (*p* < 0.01)	
incubation of washed platelets with SARS-CoV-2 (supernatant of SARS-CoV-2 infected Vero E6 cells) (1 h; 37 °C)	no agonist/flow cytometry/ELISA	increased platelet HMGB1 expression, generation of HMGB1+ microparticles, release of vWF and sCD62 (*p* < 0.0001)	Maugeri et al. [104]
incubation of washed platelets with spike protein S1 subunit (30 ng/mL)	no agonist/LTA aggregation	increased platelet aggregation (*p* < 0.001)	
incubation of PRP with spike protein or with RBD (0.5–2 µg/mL, 30–120 min)	no agonist/flow cytometry	increase in P selectin expression and GP IIbIIIa activation (*p*< 0.0001)	Cano-Mendez et al. [103]
	ADP (2 µM), collagen (2 µM), and epinephrine (10 µM)/LTA aggregation	induction of collagen-stimulated platelet aggregation (*p* < 0.0001) with no effect on ADP- or epinephrine-stimulated aggregation (NS)	
	plasma (supernatant from PRP)/ELISA	release of interleukin 6, interleukin 8, P selectin, and soluble fraction of CD40 ligand (sCD40L) (*p* < 0.05)	
incubation of washed platelets with spike protein (0.001–1 μg/mL, 15 min)	no agonist/aggregation with using flow cytometry	induced platelet aggregation in dose-dependent manner (*p* < 0.05 or less), increased expression of P selectin, CD40L, fibrinogen binding (*p* < 0.05)	Li et al. [81]
incubation of PRP with spike protein (0.001–10 μg/mL µg/mL)	collagen (0–20 µg/mL)/luminescence	increased with spike protein only but decreased for collagen-induced ATP release from platelets (*p* < 0.05)	
incubation of whole blood with spike protein (0.01–1 μg/mL)	no agonist/flow cytometry	induced platelet–monocyte aggregates (*p* < 0.05)	
incubation of washed platelets with wild-type, Alpha, Delta, Omicron spike protein (5 μg/mL)	collagen (0.2 or 0.5 μg/mL), TRAP (0.5 or 1 μM), ADP (5 μM)/LTA aggregation	no effects on platelet aggregation (NS)	Kusudo et al. [106]
	no agonist or TRAP (1 μM)/flow cytometry	no changes in P selectin expression (NS)	
incubation of whole blood with wild-type, Alpha, Delta, Omicron spike protein (5 μg/mL)	no agonist or TRAP (1 μM)/flow cytometry	no changes in GP IIbIIIa activation (PAC-1 binding) (NS)	
	no agonist/automatic hematology analyzer	no changes in platelet count and MPV independently of spike protein variant (NS)	
washed platelets in presence of 0.2, 2, and 20 µg/mL spike protein	fibronectin or collagen/adhesion	spike protein directly binds to platelet surface changes in morphology of platelets at a molecular level (*p* < 0.05); deformation of platelets itself does not always alter their intracellular signaling or induces activation but rather predispose platelets to be primed for the activation upon further stimuli	Kuhn et al. [107]
whole blood incubated with spike protein (2 μg/mL, 15 min)	collagen (0.5 µg/mL), ADP (2 µM), or TRAP (2 µM)/aggregation (Multiplate^®^)	significant reduction in ADP- and collagen induced aggregation (*p* < 0.001), no effect for TRAP (NS)	Luzak et al. [1]
	no agonist, collagen (2 µg/mL), ADP (2 µM), or TRAP (2 µM)/flow cytometry	significant reduction in PAC-1 binding for ADP-stimulated platelets (*p* < 0.01), no effect for non-stimulated platelets, collagen, and TRAP (NS); no effect for P selectin expression and fibrinogen binding (NS)	
	ristocetin (2 mg/mL) or TRAP (10 µM)/flow cytometry	increased vWF binding to ristocetin-treated platelets (*p* < 0.05); no effect for TRAP (NS)	
	fibrinogen or vWF/adhesion under flow (20 or 40 dyne/cm^2^)	unaltered adhesion for fibrinogen (NS) with a tendency to a higher level for vWF (*p* = 0.07)	
incubation of PRP with spike protein (2 μg/mL, 15 min)	collagen (1 µg/mL) and ADP (2 µmol/l)/LTA aggregation	no effect independently on agonist (NS)	
	thrombin (1 U/mL)/clot retraction	enhanced clot retraction (*p* < 0.05)	
incubation of washed platelets with spike protein (2 μg/mL, 15 min)	collagen (1 µg/mL) and ADP (2 µmol/L), thrombin (0.1 U/mL)/LTA aggregation	increased collagen-induced aggregation (*p* < 0.01), no effect for ADP and thrombin (NS)	

HMGB1—high mobility group box 1; RBD—receptor binding domain; PRP—platelet rich plasma; PFU—Plaque forming units; vWF—von Willebrand factor; MPV—mean platelet volume4.4. Possible targets in blood platelets for SARS-CoV-2.

It was reported that platelets express receptors specialized in microbe recognition [108]. Potential targets for SARS-CoV-2 in platelets include heparan sulfate proteoglycans (HSPGs), C-type lectin receptors (CLRs), Toll-like receptors (TLRs), and extracellular matrix metalloproteinase (CD147) [109]. The main cell entry receptor for SARS-CoV-2, ACE2 (angiotensin), is generally regarded as being absent from blood platelets [26,55,58,71,110]. CD147, also known as basigin (BSG) or EMMPRIN, a receptor constitutively expressed on a large fraction of human platelets, has been described more as a coreceptor, i.e., an attachment cofactor, involved in SARS-CoV-2 infection than as an important receptor needed for virus interaction with platelets. CD147 was found to be involved in the spike protein-dependent activation of platelets, the aggregation and release of granules, the release of soluble P selectin, and the release of HMGB1+ microplatelets [104]. Carnevale et al. found the SARS-CoV-2 spike protein to favor platelet activation by directly interacting with platelet TLR4 and demonstrated that the spike protein per se does not promote platelet activation but amplifies the platelet response to the agonists via interaction with TLR4 [111]. Alternative receptors that could bind SARS-CoV-2 are the integrins GPIIbIIIa or GPIb [81,84,112,113]. In the presence of antibodies against SARS-CoV-2 or cross-reacting antibodies against more prevalent coronaviruses that cause minor cold symptoms, viruses can also activate platelets through indirect interactions with FcRIIA [114].

An interesting proposal by Scheim et al. [115,116] is that SARS-CoV-2’s initial attachment to host cells is through the binding of its spike protein to sialylated glycans, containing the monosaccharide sialic acid, on the cell surface. As red blood cells, platelets, and endothelial cells are all densely coated with sialic acid molecules, such binding can result in blood cell aggregation, microvascular occlusion, and vascular damage that underlie the hypoxia, blood clotting, and related morbidities of severe COVID-19 [116].

## 5. Prolongated Platelet Hyperreactivity After COVID-19

Similarly to the thrombotic complications observed during COVID-19, patients with post-acute sequelae of COVID-19 (PASC) demonstrate dysregulated responses in platelets and coagulation in plasma, likely caused by circulating molecules that promote thrombosis [117]. PASC is a recognized multisystemic condition characterized by persistent symptoms four weeks beyond the initial SARS-CoV-2 infection. Patients with PASC develop or have persistent symptoms for several weeks after recovery, and this phenomenon was labeled “long COVID”. The platelet hyperactivity persisted for at least 40 days even after acute inflammation subsided in most patients with COVID-19, regardless of disease severity [28]. Higher activation of circulating platelets was demonstrated in adults one year after COVID-19, indicated by elevated CD62P and CD36 levels, which may be explained by immune dysregulation and persistent inflammation triggered by the initial infection [118]. In children with long COVID, the circulating platelets had significantly increased P selectin expression compared with healthy controls [119]. In addition to platelet activation, this observation also confirms previous findings regarding the activation of hemostasis and inflammation processes in children with long COVID. Factors that trigger such activation are still unknown, although adult studies suggest that viral persistence and endothelial inflammation may play a role [119]. Nicolai et al. propose that thromboinflammation in long COVID may be associated with (i) lasting structural changes, most prominently endothelial damage, caused during initial infection, (ii) a persistent viral reservoir, and iii) immunopathology driven by a misguided immune system [120]. They outline the necessity for large, well characterized clinical cohorts and mechanistic studies to clarify the contribution of thromboinflammation to long COVID [120].

## 6. Concluding Remarks

Despite the high variability in the results regarding the activation and reactivity of platelet in COVID-19 or in the presence of SARS-CoV-2, it seems certain that platelet dysfunction plays an important role in this disorder. While most publications, typically those using isolated platelets, report platelet hyperreactivity, it should be noted that differences in platelet function are observed in various phases of the disease. Indeed, some publications report a decrease in platelet reactivity in COVID-19. Furthermore, many publications do not precisely describe the selection of the study group, which may have a considerable influence on the test results.

Changes in platelet function could result from their direct interaction with SARS-CoV-2 or its constituents; however, there is currently a lack of consensus with regards to specific targets in platelets for the virus. Importantly, platelet function is also influenced by other cells and plasma components, including antibodies and inflammatory mediators. Generally, higher activity and lower reactivity are observed with significant decreases in platelet count, as well as the opposite, where platelet count remains the same or increases. In our opinion, the best choices for evaluating platelet function in COVID-19 are flow cytometry, the measurement of soluble markers of platelet activation in plasma, and platelet aggregation in PRP (LTA variant). Flow cytometry is a reliable technique characterized by the possibility to measure multiple parameters (markers) based on a relatively small amount of biological material (blood, PRP) and, what is important, it enables the evaluation of both basal platelet activation and reactivity after using a wide panel of agonists. The determination of soluble markers of platelet activation in plasma is a simple, well-standardized method allowing for the use of frozen samples. The LTA variant of measuring platelet aggregation, in turn, is still regarded as a golden standard for a relatively simple assessment of platelet reactivity. Other tests, such as whole blood aggregometry (WBA), PFA, T-TAS, and TEG/TEM, can be used when platelet count is in the normal range. 

## Figures and Tables

**Table 1:** Platelet-related parameters calculated on the basis of blood cell counts.

Parameter	Full Name of Parameter	Parameter Description
AISI	aggregate index of systemic inflammation	neutrohphil × platelet × monocyte/lymphocyte ratio
MII-1	multi-inflammtory index-1	platelet/lymphocyte × CRP (PLR × CRP)
MII-3	multi-inflammatory index-3	neutrohphil × platelet/lymphocyte × CRP (SII × CRP)
MPVLR	-	mean platelet volume/lymphocyte ratio
NPR	-	neutrophile/platelet ratio
PLR	-	platelet/lymphocyte ratio
SII	systemic immune-inflammation index	neutrohphil × platelet/lymphocyte ratio

CRP—C-reactive protein; MPV—mean platelet volume.
